# Prenatal Diagnosis of Thoracoschisis and Review of Literature

**DOI:** 10.1155/2017/9821213

**Published:** 2017-11-16

**Authors:** Hasaruddin R. Hanafi, Zahar A. Zakaria

**Affiliations:** ^1^Paediatric Department, Hospital Kemaman, Terengganu, Malaysia; ^2^Obstetrics & Gynaecology Department, Hospital Kemaman, Terengganu, Malaysia

## Abstract

Thoracoschisis is a rare congenital malformation characterized by herniation of the abdominal content through a defect in the thorax. There are previously 12 reported cases, most discussing the postnatal findings and management. Here we describe a case of left thoracoschisis with associated upper limb abnormality which was diagnosed antenatally with the aid of 3D ultrasound.

## 1. Introduction

Thoracoschisis is a very rare congenital anomaly characterized by the herniation of intra-abdominal organs through a thoracic wall defect. It may be an isolated malformation or associated other abnormalities including limb and thoracoabdominal wall defect, forming part of a complex malformation, the limb body wall complex (LBWC) [1]. Currently, there are twelve reported cases and all, except one, were diagnosed after delivery (Table 1). Here we are describing a prenatally diagnosed thoracoschisis associated with limb abnormality.

## 2. Case Report

A 27-year-old lady in her 2nd pregnancy was referred to the Obstetrics & Gynaecology Department, Hospital Kemaman, at 32-week pregnancy for further assessment of disparity between the fundal height measurement and gestational age, noted during her routine antenatal care. The previous pregnancy was uneventful, resulting in a birth of a healthy 2.8 kg baby at term and the patient had no significant medical problem.

**Figure 1:**
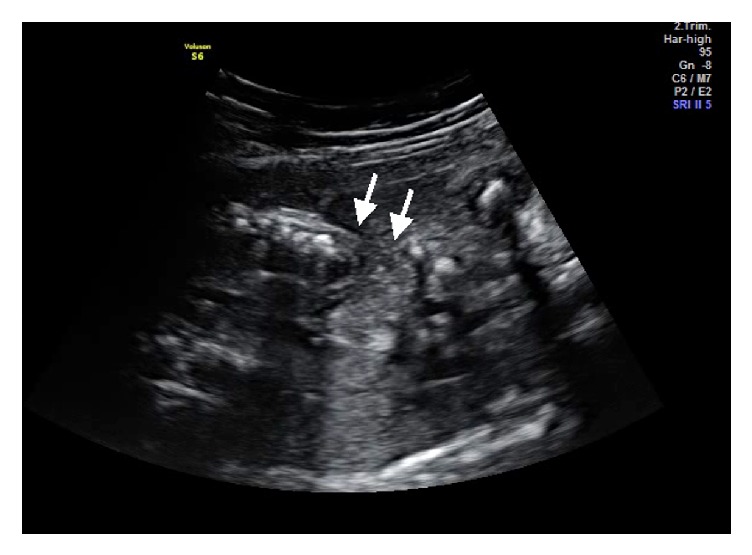
Rib defect on 2D mode (arrow pointing to the 3rd and 4th rib).

**Figure 2:**
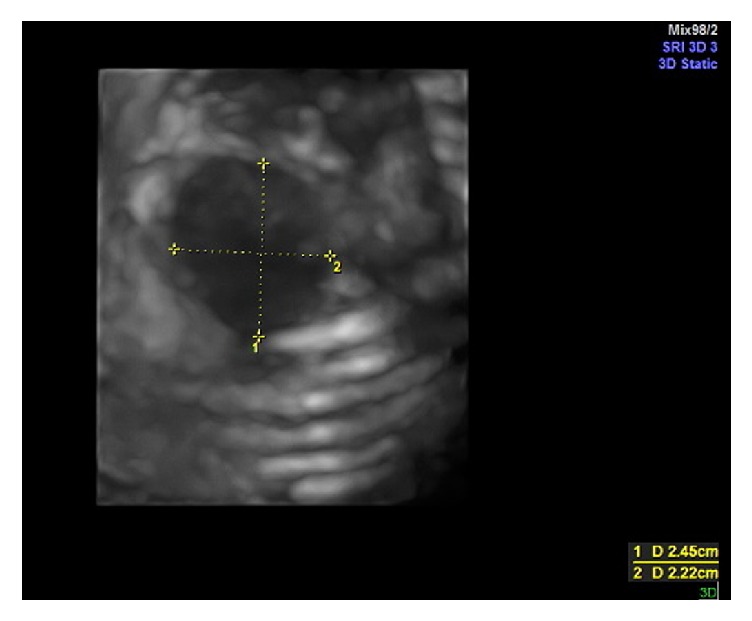
Rib defect and hernia orifice on 3D mode.

Ultrasound examination was performed using a Voluson S6 (GE Healthcare Ultrasound, Milwaukee, WI, USA) equipped with a 3–5 MHz curvilinear 3D abdominal probe which showed a single fetus with parameters corresponding to 32-week gestation with normal liquor volume. The fetal heart was pushed to the right with the left hemithorax filled with fetal liver and part of the small intestine. There was a defect in the left anterolateral part of the 3rd and 4th rib which was identified on 2D mode, with herniation of the stomach, intestine, and part of the left lobe of the liver (Figure 1). Using the 3D ultrasound probe, a volume was acquired and a mixture of surface mode and transparent maximal rendering was used to show the bony ribs, the absence of the anterior part of the involved ribs, and also the size of the defect on the chest (Figure 2). There was also absence of left radius with concurrent curved ulna. Other structures were normal. The subsequent visits to our centre revealed slowing of the fetal growth but no changes seen on the thoracic defect or the herniated organs.

The delivery was planned to be completed at a tertiary centre with paediatric surgical service but the patient went into spontaneous labour at 36 weeks of gestation. It was an uncomplicated event resulting in delivery of a 1500 gm boy. The neonate had Apgar score of 9 and 10 at the 1st and 5th minutes of life, respectively, with good, spontaneous breathing effort.

**Figure 3:**
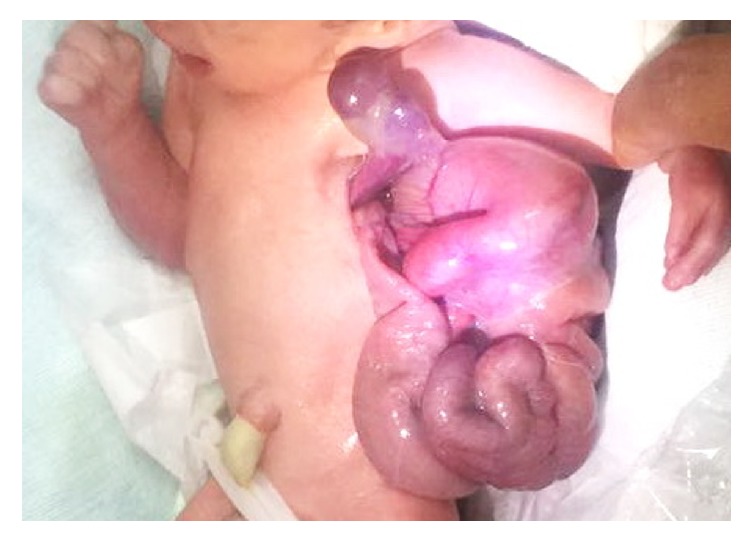
Visceral herniation.

**Figure 4:**
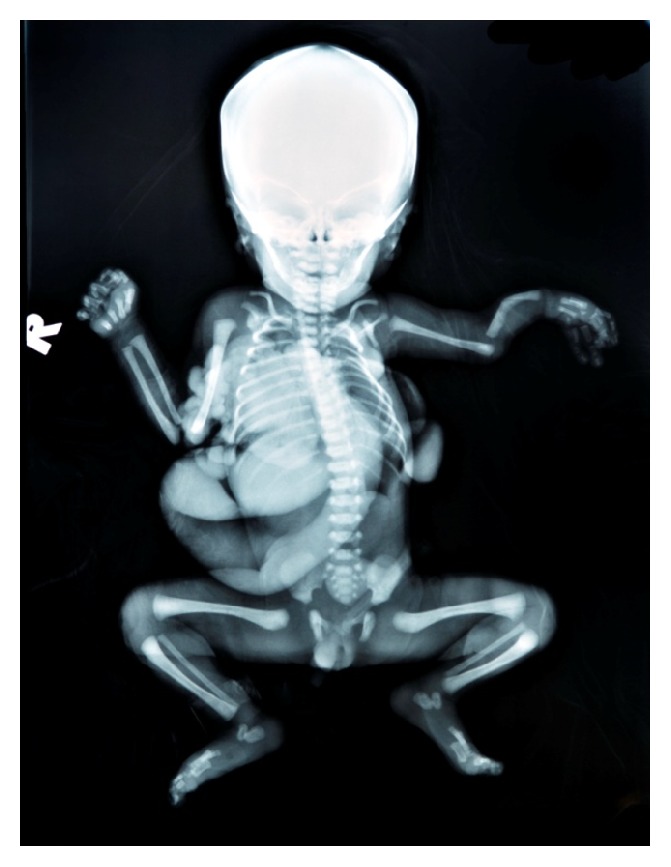
Skeletal radiograph.

Examination after the delivery showed herniation of the stomach, small intestine, part of the left lobe of the liver, and spleen through a 3 × 3 cm defect on left thorax, at the anterior axillary line (Figure 3). There was polydactyly with fused interdigits of the left hand, overlapping fingers of the right hand, bilateral rocker-bottom foot, and bilateral cryptorchidism. A nasogastric tube was inserted with tip located outside the thoracic cavity while an echocardiogram showed normal heart structures. Radiographic examination revealed absence of anterior part of left 3rd and 4th rib with left type IV (Bayne and Klug classification) [2] radial aplasia with bent and short radially bowed ulna (Figure 4).

In the first few hours of life, the baby was stable and breathing on his own while the preparation is made for transfer to a tertiary hospital with paediatric surgical expertise. Despite the initial promising outcome, the boy developed respiratory distress 12 hours after delivery requiring intubation and mechanical ventilation. He was on the respiratory support for the next 2 days but the condition worsened with evidence of sepsis and passed away at day 3 of life. The parents declined postmortem examination.

## 3. Discussion

Thoracoschisis is a very rare congenital abnormality characterized by the herniation of intra-abdominal organs through a chest wall defect. Currently, only twelve cases have been described in the literatures and most had associated abnormality especially the diaphragm and limbs.

In was unfortunate that, in our case, no postmortem examination could be performed to determine the state of the diaphragm but in other reported cases, seven had confirmed diaphragmatic hernia while five others were intact but located superior to the thoracoschisis opening. Five cases had the herniation occurring in the intercostals space while seven had associated rib agenesis or aplasia, which involved up to 4 ribs (Table 1). All, except one case, had evisceration of part of the liver, with or without stomach and/or intestinal involvement. Four cases were isolated thoracoschisis without limb abnormality, while the rest had associated abnormality, ranging from agenesis of the upper to minor defect such as a single anomaly.

Aetiology of thoracoschisis is unclear but some authors consider it as part of limb body wall complex (LBWC) spectrum as it is mostly associated with some forms of limb defect. The proposed theories on the cause of LBWC include early amniotic sac rupture and formation of amniotic bands which entrap and disrupt limb formation, abnormal embryonic folding, and vascular abnormality causing the internal malformations with the abnormalities of the limbs [1, 15, 16]. The mild limb defect in our case, with no evidence of amniotic band effect, suggests the vascular malformation as the most probable aetiology. Van Allen et al. had demonstrated the presence of abnormal vasculatures in radial aplasia, suggesting the role of abnormal vascular development causing the limb abnormality [17]. It is possible that vascular abnormality of the left side leads to the development of the thoracic defect and the left upper limb abnormalities seen in our case.

LBWC had been diagnosed as early as first trimester and there are possibilities that some of these, including those that underwent termination of pregnancy, were associated with thoracoschisis [18]. Of the reported case of the thoracoschisis, most were diagnosed after birth or diagnosed as gastroschisis on prenatal ultrasound [4, 11]. This is the first reported case of prenatally diagnosed thoracoschisis, using the 2D and 3D ultrasound demonstrating the herniation, the location, and size of the defect. We believe that the abnormality can be confidently diagnosed earlier in pregnancy and could help streamlining the subsequent management after the delivery.

## Figures and Tables

**Table 1:** Reported cases of thoracoschisis.

	Reference	Gender	Defect location	Content	Associated anomalies	Diagnosis
(1)	Davies et al., 1977 [3]	Female	Left third intercostal	Left liver lobe, stomach, transverse colon	No left forearm, syndactyly, dextrocardia	Postnatal

(2)	Bamforth et al., 1992 [4]	Female	Left sixth rib	Left liver lobe	Left Poland anomaly, scapula hypoplastic, no humerus, no ulna, no radius, dextrocardia	Postnatal

(3)	Derbent and Balci, 2001 [5]	Female	Right second to fourth rib	Liver intestine	LBWC, other deformations not able to be defined	Postnatal^*∗*^

(4)	Biri et al., 2006 [6]	Female	Left (unspecified location)	Left liver lobe	Left forearm agenesis, right arm-hand agenesis	Postnatal

(5)	Karaman et al., 2011 [7]	Male	Left eighth intercostal	Liver, transverse colon, omentum	None	Postnatal

(6)	Bhattacharyya et al., 2012 [8]	Female	Right (absence of ribs)?	Riedel liver lobe, stomach, small intestine	Agenesis of the right upper limb, right upper quadrant abdominal wall defect	Postnatal^*∗*^

(7)	Eck et al., 2015 [9]	Male	Right fifth–eight ribs	Liver, intestine, omentum	Right fourth digit abnormality	Postnatal

(8)	McKay et al., 2015 [10]	Female	Left eighth intercostal	Riedel liver lobe, omentum	Positional deformity of left hand & palmar contractures of the fingers	Postnatal

(9)	Seleim et al., 2015 [11]	Male	Left 4th intercostal	Riedel liver lobe, stomach, intestine	None	Postnatal

(10)	Travers et al., 2016 [12]	Female	Left rib aplasia (unspecified location)	Mesenchymal hamartoma	None	Postnatal

(11)	de Grijs et al., 2017 [13]	Female	Left of fourth–sixth ribs	Liver, stomach, transverse colon	None	Antenatal

(12)	Vujovic et al., 2017 [14]	Female	Right first intercostal	Riedel liver lobe	Hypoplasia of the right arm and incomplete hand duplication	Postnatal

(13)	Current case	Male	Left third and fourth ribs	Left liver lobe, stomach, spleen, intestine	Radial aplasia, unilateral polydactyly and syndactyly	Antenatal

^*∗*^Diagnosed prenatally as gastroschisis.

## References

[1] Van Allen M. I., Curry C., Gallagher L. (1987). Limb body wall complex: I. Pathogenesis. *American Journal of Medical Genetics*.

[2] Bayne L. G., Klug M. S. (1987). Long-term review of the surgical treatment of radial deficiencies. *Journal of Hand Surgery*.

[3] Davies M. R. Q., Rode H., Cywes S. (1977). "Thoracoschisis" associated with an ipsilateral distal phocomelia and an anterolateral diaphragmatic hernia-A case report. *Journal of Pediatric Surgery*.

[4] Bamforth J. S., Fabian C., Machin G., Honore L. (1992). Poland anomaly with a limb body wall disruption defect: Case report and review. *American Journal of Medical Genetics*.

[5] Derbent M., Balci S. (2001). Thoracoschisis associated with diaphragmatic hernia in a 31-week-old stillbirth. *The Turkish Journal of Pediatrics*.

[6] Biri A., Korucuoglu U., Turp A., Karaoguz M., Himmetoglu O., Balci S. (2006). A new syndrome with prenatally diagnosed hiatal hernia and extremities' agenesis: case report. *Genetic Counseling*.

[7] Karaman İ., Karaman A., Erdoğan D., Çavuşoğlu Y. H., Özgüner İ. F. (2011). The first male with thoracoschisis: case report and review of the literature. *Journal of Pediatric Surgery*.

[8] Bhattacharyya N., Gogoi M., Deuri P. (2012). Thoracoschisis with limb agenesis. *Journal of Indian Association of Pediatric Surgeons*.

[9] Eck D. L., Maryak B. N., Poulos N. D., Tepas J. J., Felema G. G., Robie D. K. (2015). Thoracoschisis: Case report and review of the literature. *Annals of Pediatric Surgery*.

[10] McKay J. D., Parker C. M., Loewen J. (2015). Thoracoschisis: A Case Report and Review of Literature. *Fetal and Pediatric Pathology*.

[11] Seleim H., ElFiky M., Fares A., Elbarbary M. (2015). Isolated Thoracoschisis: Case Report and Review of Literature. *European Journal of Pediatric Surgery Reports*.

[12] Travers C. P., Hamm J. A., Cleveland S., Chen M. K., Anderson S., Philips J. B. (2016). Thoracoschisis secondary to a mesenchymal hamartoma associated with diaphragmatic eventration. *Case Reports in Perinatal Medicine*.

[13] de Grijs D., Israelyan N., DeVore G. R., Chen S. C., Shekherdimian S. (2017). An unusual occurrence of isolated thoracoschisis. *Journal of Pediatric Surgery Case Reports*.

[14] Vujovic D., Sretenovic A., Raicevic M. (2017). Thoracoschisis associated with Limb Body Wall Complex. *APSP Journal of Case Reports*.

[15] Torpin R. (1965). Amniochorionic mesoblastic fibrous strings and amnionic bands. Associated constricting fetal malformations or fetal death. *American Journal of Obstetrics & Gynecology*.

[16] Streeter G. L. (1930). Focal deficiencies in fetal tissues and their relation to intrauterine amputations. *Contributions to Embryology*.

[17] Van Allen M. I., Hoyme H. E., Jones K. L. (1982). Vascular pathogenesis of limb defects. I. Radial artery anatomy in radial aplasia. *Journal of Pediatrics*.

[18] Murphy A., Platt L. D. (2011). First-Trimester diagnosis of body stalk anomaly using 2-and 3-dimensional sonography. *Journal of Ultrasound in Medicine*.

